# Macular Microvascular Modifications in Progressive Lamellar Macular Holes

**DOI:** 10.3390/diagnostics11091717

**Published:** 2021-09-19

**Authors:** Fiammetta Catania, Davide Allegrini, Alessandra Nembri, Filippo Confalonieri, Piero Zollet, Emanuele Crincoli, Mario R Romano

**Affiliations:** 1 Department of Biomedical Sciences, Humanitas University, Via Rita Levi Montalcini 4, 20090 Milan, Italy; filippo.confalonieri01@gmail.com (F.C.); zolletpiero@gmail.com (P.Z.); mario.romano.md@gmail.com (M.R.R.); 2 Department of Ophthalmology, Humanitas Gavazzeni-Castelli, 24128 Bergamo, Italy; davideallegrini@yahoo.it (D.A.); alessandra.nembri@gmail.com (A.N.); 3 Ophthalmology Unit, “Fondazione Policlinico Universitario A. Gemelli IRCCS”, 00168 Rome, Italy; emanuelecrincoli1@gmail.com; 4 Department of Ophthalmology, Catholic University of “Sacro Cuore”, 00168 Rome, Italy

**Keywords:** lamellar macular hole, oct angiography, neurovascular unit, retinal microvasculature, Muller cells

## Abstract

Lamellar macular holes (LMHs) may show morphological and functional deterioration over time, yet no definite prognostic factor for progression has been identified. Since neurovascular retinal unit impairment may take part in neurodegeneration, we compare progressive LMHs to stable ones in optical coherence tomography (OCT) angiography parameters. Methods: OCT B scans of eyes with LMH were analyzed to detect the presence of tissue loss (TL) over time, allowing us to identify a TL group and a stable (ST) group (14 patients each). The best corrected visual acuity (BCVA) at each considered imaging time point was collected. Lastly, patients underwent macular OCT angiography. Results: BCVA at last follow up was significantly reduced in the TL group compared to both the ST group and TL group baseline assessment. SCP foveal vessel density (VD), SCP and deep capillary plexus (DCP) perfusion density (PD) and parafoveal PD were lower in the TL group. Linear correlations between quantitative TL over time and parafoveal PD in SCP and between the speed of TL and BCVA variation during follow up were also detected. Conclusions: TL in LMHs is associated with both OCT angiography modifications and BCVA deterioration over time. We suggest these findings to be a manifestation of foveal Muller cell impairment in progressive LMHs.

## 1. Introduction

Lamellar macular hole (LMH) is a vitreoretinal interface disorder affecting 1.1 to 3.6% of the general population, mainly within the range of 50–70 years of age [1,2,3]. No significant gender prevalence and a possible protective role of diabetes are reported in the literature [3]. The formation of anterior–posterior traction forces exerted on the fovea by posterior vitreous detachment (PVD) has long been recognized as a fundamental step in the development of the condition [4,5]. Despite the common pathogenetic pathway, two different types of LMH can be distinguished on the basis of morphological and functional characteristics [6]. The first, currently known as epiretinal membrane (ERM) foveoschisis, is characterized by the presence of a contractile ERM, foveoschisis at the level of Henle fiber layer (HFL), microcystic spaces in the inner nuclear layer (INL), retinal thickening and retinal wrinkling. The second, which is referred to as “true” LMH, is identified through the presence of irregular foveal contour, foveal cavities with undermined edges and signs of foveal tissue loss. Simultaneous presence of epiretinal proliferation, foveal bump and ellipsoid line disruption is frequent as well [7]. In addition to anatomical characteristics, LMHs are characterized by a more pronounced visual acuity (VA) loss, retinal pigmented epithelium (RPE) damage, and poorer response to surgical treatment compared to ERM foveoschisis [6,7,8]. Moreover, a proportion of patients with LMHs manifest severe visual impairment and/or show anatomical signs of progressive tissue loss on optical coherence tomography [8,9]. The biggest limit of this classification of LMHs is the inability to predict functional prognosis due to the fact that no morphological parameter has been clearly correlated to the progression of the disease to the present date [10]. The development of a progression independently from the dynamics of PVD has raised attention on the contribution of other mechanisms in alimenting the pathological process [11,12]. In particular, since neurovascular retinal unit impairment plays a decisive role in neurodegeneration in various neurological and retinal diseases, we hypothesize that retinal microvascular modifications could be involved in the progression of LMHs [11,13,14,15]. The aim of the study is therefore to assess differences in OCTA parameters in progressive degenerative LMHs compared to stable ones.

## 2. Materials and Methods

**Figure 1:**
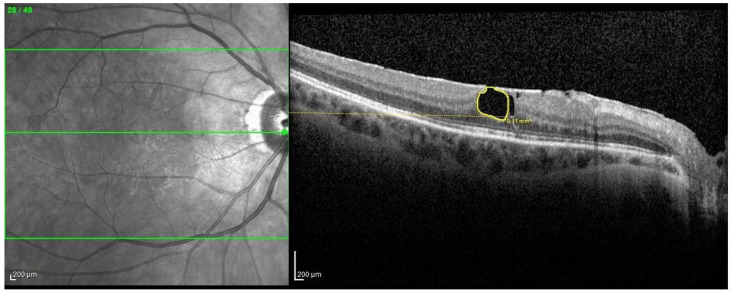
Tissue-loss contouring with automatic gap area calculation in a patient from TL group.

The study was designed as a monocentric case–control study analyzing patients diagnosed with and followed up for LMH from January 2017 to April 2021 in the Ophthalmology department of Humanitas Castelli University Hospital. Patients were retrospectively selected from the OCT database according to the following inclusion criteria: presence of LMH, availability of at least two high-quality OCT examinations with a minimum interval of 6 months and availability of anamnestic and ophthalmic data, including distance best corrected visual acuity (BCVA) and intraocular pressure (IOP). Exclusion criteria were previous vitreoretinal surgery for the treatment of LMH or other conditions, diagnosis of cataract, diabetic retinopathy, previous vein occlusion, choroidal neovascularization from every cause, moderate to severe dry age-related macular degeneration (AMD), glaucoma, high myopia (> 6 diopters of refractive error) and cystoid macular edema (CME). LMH was defined in accordance with the recent expert consensus definition as a partial-thickness defect in the inner fovea characterized by a foveal cavity with undermined edges, inconstant presence of epiretinal proliferation, frequent disruption of the outer retina and in some cases, the appearance of a central bump of presumably spared foveal tissue [16]. OCT examination at all the considered time points had to include high resolution volumetric assessment of the central retinal structures, with a scanning dimension of at least 20° × 15° centered on the fovea and an interscan distance ≤60 μm. Patients were subsequently divided into 2 groups according to the presence of tissue loss from baseline to follow-up examination, defining a tissue loss group (TL group) and a stable group (ST group). Tissue loss (TL) was independently assessed by two expert graders by analyzing digital measurements of the area of retinal gap derived from manual contouring (see Figure 1). Tissue loss for a specific section was determined by assessing algebraic subtraction between the gap area at baseline and the gap area at follow-up examination. Quantitative analysis of TL was performed by summing TLs detected in the 3 most involved sections from each patient. A TL of at least 0.02 mm^2^ was defined as a cut off for enrollment in TL group. Moreover, the interval between examinations was considered in order to calculate the speed of tissue loss during the analyzed period.

**Figure 2:**
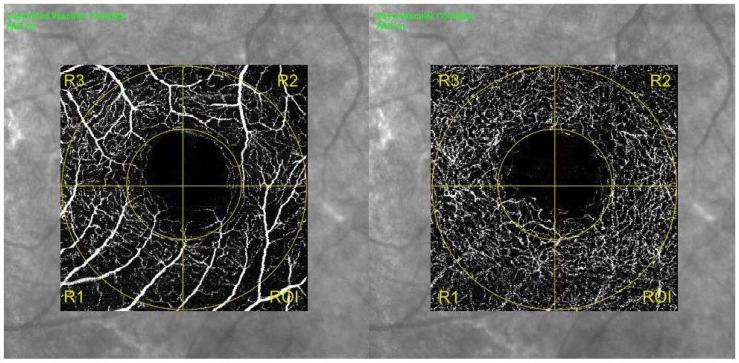
Standard grid applied for foveal and parafoveal zone isolation in SCP (left side of the image) and DCP (right side of the image). ROI indicates the quadrant matching with the zone of tissue loss in B scan images of TL group. Analysis of VD and PD detected a lower parafoveal PD in SCP of ROI regions compared to the remaining 3. DCP = deep capillary plexus; PD = perfusion density; ROI = region of interest; SCP = superficial capillary plexus; VD = vessel density.

Patients from both groups were summoned to undergo BCVA assessment and OCT examination including structural OCT and OCT angiography (OCTA). Acquisitions were performed with Spectralis SD OCT (Spectralis HRAþOCT, software version 5.4.7.0; Heidelberg Engineering, Inc., Heidelberg, Germany). Spectralis OCT uses a dual beam SD OCT, a confocal laser-scanning ophthalmoscope with a wavelength of 870 nm, and an infrared reference image to obtain images of ocular microstructures with an acquisition rate of 40,000 A-scans per second. It also incorporates a real-time eye-tracking system that couples confocal laser-scanning ophthalmoscopy and SD OCT scanners to adjust for eye movements. B scan examinations were performed using the follow up mode setting baseline examination as a reference. OCTA foveal acquisition consisted of a 15 × 15° angle with a lateral resolution of 5.7 µm/pixel, resulting in a retinal section of 2.9 × 2.9 mm. Only images with a Q score > 15 were included in the analysis. An image of the superficial capillary plexus (SCP) and deep capillary plexus (DCP) was generated using automated layer segmentation, corrected by manual readjustments of the segmentation lines. The boundaries for segmentation were from the inner limiting membrane (ILM) to the outer border of the inner plexiform layer (IPL) for the SCP, and from the outer border of the IPL to the outer border of the outer plexiform layer (OPL) for the DCP. Manual readjustment was needed in 2 cases only. En-face images were processed using a customized MATLAB v7.10 (Mathworks, Inc.) software combining a global threshold, Hessian filter, and adaptive threshold to generate binary vessel maps, which were used to calculate quantitative indices of blood flow and vessel density. Vessel density (VD) was expressed in a percentage and derived from the ratio of the total vessel area (all white pixels, defined as pixels with a ratio value between 0.7 and 1.0) to the total area of the analyzed region (size of the image in pixels). Perfusion density (PD) was defined as the percentage of the total analyzed area occupied by perfused vessels, which was derived from the ratio of the total perfused vessel area to the total area of the analyzed region. Perfused vessels were identified as vessels characterized by decorrelation on different frames. En-face angiograms were further elaborated, superimposing a grid delimiting 4 parafoveal regions and 4 foveal regions on retinal angiograms of SCP and DCP. Specifically, a circular area of 1 mm diameter centered on the fovea was considered as a foveal region. The parafoveal region consisted of a ring area delimitated internally by the limit of the foveal region and externally by a circle of 2.9 mm diameter centered on the fovea. The retinal angiogram quadrant colocalizing with tissue loss was named the region of interest (ROI), while the two adjacent quadrants were named R1 and R2 and the opposite quadrant was named region R3 (Figure 2). VD and PD were calculated for both plexuses in each region. The capillary free zone (CFZ) perimeter and CFZ area were automatically generated by OCT software after manual contouring of the border of the vascularized zone by two expert graders. CFZ circularity was measured using the following equation: circularity = 4πA/P^2^, where A is the area and P is the perimeter. Using this equation, as the shape becomes less round or less smooth, the circularity approaches zero.

### Statistical Analysis

Statistical analysis was conducted using SPSS software (IBM SPSS Statistics 26.0). Normality of the distribution for quantitative variables was evaluated using a Shapiro–Wilk test. Normally distributed variables were described using the mean and standard deviation, while for the description of non-normally distributed variables, the median and interquartile range (IQR) were adopted. Qualitative variables were described as the number of cases over the total and percentage. The agreement between graders in TL quantification was assessed using an intraclass correlation coefficient (ICC). A Wilcoxon signed rank test was used for univariate analysis of BCVA and IOP variations over time in each of the two study groups. Univariate comparison of quantitative variables between the TL group and ST group was performed using a Mann–Whitney test or two-tailed independent samples T test as appropriate. For quantitative variables, X^2^ or Fisher’s exact test with post hoc corrections were used instead. Spearman’s correlation was adopted to assess linear correlations between quantitative variables. A *p* value < 0.05 was considered as statistically significant.

## 3. Results

A total of 28 patients were included in the analysis, with 14 patients belonging to the TL group and 14 to the ST group. The median age of the total population was 73.7 (7.1) years, and 10 patients (35.7%) were male. No differences in age or sex composition were detected between the two groups. Even though patients from the TL group showed a slightly higher prevalence of cerebral vasculopathy, diabetes mellitus and systemic arterial hypertension, none of these differences reached statistical significance (see Table 1). BCVA at baseline was similar in the two groups, with a median value of 0.9 (0.12) decimals in the TL group and 0.9 (0.05) decimals in the ST group (*p* = 0.23). The groups also did not differ significantly as concerns spherical equivalent (SE) (*p* = 0.75) and prevalence of pseudophakic patients at baseline (*p* = 0.68) (Table 1). Follow-up duration in the overall population was 2.25 (0.49) years and did not differ significantly in the two groups (0.88). Nevertheless, BCVA at the last follow up was statistically significantly higher in ST group compared to the TL group, with a median value of 0.9 (0.1) decimals and 0.78 (0.09) decimals, respectively. Moreover, paired samples analysis revealed a statistically significant loss in BCVA during the follow-up period in the TL group (*p* = 0.001) which was not detected in ST group (*p* = 0.056). Both groups showed a median IOP value within a normal range both at baseline and at follow-up examination and no statistically significant difference was detected both between and within groups. Further functional and anamnestic details are provided in Table 1.

In the TL group, the median amount of tissue loss was 0.09 (0.055) mm^2^ with a median speed of tissue loss of 0.04 (0.02) mm^2^/year. ICC showed a good correlation between graders in the quantitative assessment of tissue loss (ICC = 0.863, r = 0.810). Baseline OCT B scan analysis of the total population revealed a complete PVD in 11 patients (39.28%) and an incomplete PVD in 12 patients (42.85%) with no significant difference in the two groups concerning both (*p* = 0.99 and *p* = 0.70, respectively). Four cases of lamellar hole-associated epiretinal proliferation (LHEP) were detected in the total population (14.28%). Interestingly, all of them belonged to patients from the TL group, resulting in a 28.57% prevalence of the condition. Nonetheless, this difference with the ST group did not reach statistical significance (0.098). Five out of twenty-eight patients (17.85%) presented focal foveal ellipsoid zone (EZ) disruption with no difference in prevalence in the two groups (*p* = 0.99).

**Figure 3:**
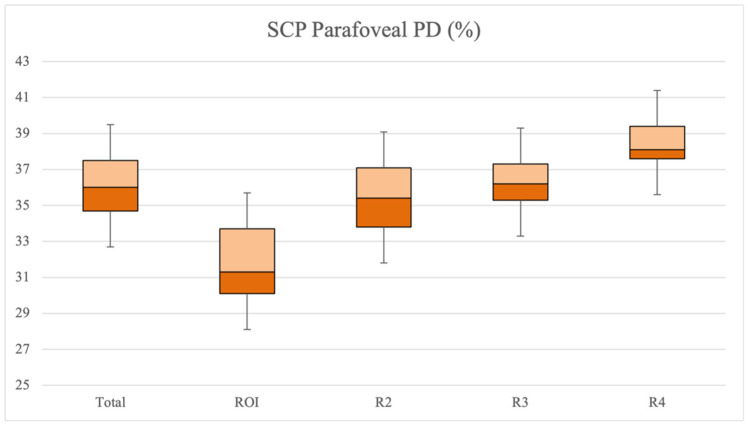
Representation of SCP parafoveal PD (%) in total parafoveal area, ROI and remaining regions in TL group. PD = perfusion density; ROI = region of interest; SCP = superficial capillary plexus; TL = tissue loss.

**Figure 4:**
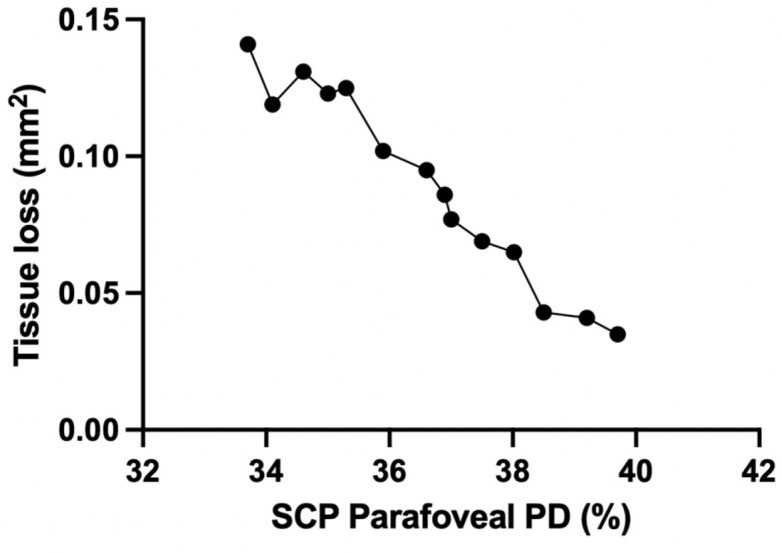
Linear correlation between SCP parafoveal PD (%) and tissue loss during the follow-up period (mm^2^) in TL group. PD = perfusion density; SCP = superficial capillary plexus; TL = tissue loss.

**Figure 5:**
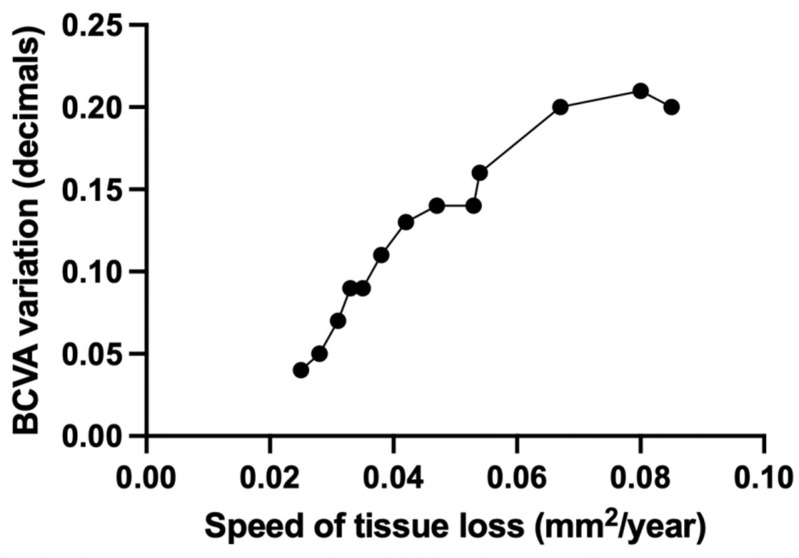
Linear correlation between speed of tissue loss (mm^2^/year) and BCVA variation during follow up period (decimals) in TL group. BCVA = best corrected visual acuity; TL = tissue loss.

OCT-A analysis of SCP showed the TL group to be characterized by a statistically significantly higher CFZ perimeter (*p* = 0.041) and CFZ area (*p* = 0.036) and lower CFZ circularity (*p* = 0.003) compared to the ST group. Moreover, both foveal VD, foveal PD and parafoveal PD in SCP were statistically significantly lower in TL group (respectively *p* = 0.012, *p* = 0.007, *p* = 0.009) (Table 2) and SCP parafoveal PD was significantly reduced in the ROI (median value of 31.3 (3.6)) compared to the other regions (*p* = 0.006) (Figure 3). In addition, a statistically significant linear correlation between the amount of tissue loss during the follow up period and the total parafoveal PD in SCP was detected in the TL group (*p* < 0.001, r = −0.9912) (Figure 4). Concerning DCP, the TL group showed a reduced CFZ circularity (*p* = 0.003) and a lower foveal PD (*p* = 0.021) and parafoveal PD (*p* = 0.005) compared to the ST group (Table 2). No significant VD and PD differences were detected between the ROI and the other regions in DCP. Lastly, speed of tissue loss had a statistically significant linear correlation with BCVA variation during follow up time (*p* < 0.001, r = 0.9901) (Figure 5).

## 4. Discussion

Neurovascular coupling, first documented by Roy and Sherrington in 1890 [16], is the ability of the central nervous system to evoke localized changes in blood flow and long-term modifications in vessel density in response to neuronal activity. Glial cells act as intermediaries between neurons and blood vessels in the control of this mechanism, owing to their ability to release vasoactive factors in response to neuronal stimuli [17,18]. The complex functional interactions between glial, neural, and vascular retinal elements can be summed up by the concept of neurovascular retinal unit. Disorders of this interplay have been demonstrated to exert a pivotal role in the pathogenesis and progression of various retinal diseases, such as glaucoma and diabetic retinopathy [11,14,15]. Our paper aimed to investigate the involvement of microvascular blood flow variations in the neurodegeneration observed in progressive LMHs. Consistently with our hypothesis, Yeo et al. [12] recently highlighted angiographic differences between LMHs and ERM foveoschisis, reporting a larger CFZ and a lower parafoveal VD in both SCP and DCP in the former and demonstrating a correlation between BCVA and foveal and parafoveal VD in LMHs which was not present in ERM foveoschisis. Recently, Dell’Omo et al. also highlighted how discrete areas of central and peripheral leakage are commonly found in eyes with LMH in fluorescein angiograms [19]. Our paper detected significant microvascular differences between anatomically progressing and stable LMHs and, most importantly, identified tissue loss as a significant functional prognostic factor in the natural history of the disease. Concerning OCT-A parameters, progressive LMHs showed a significantly increased CFZ area and perimeter in SCP angiograms and a lower CFZ circularity in both SCP and DCP. In particular, CFZ circularity mean values in both groups were lower compared to normal controls according to the literature [20]. Moreover, foveal and parafoveal PD were found to be significantly lower in both SCP and DCP in progressive compared to stable LMHs. Specifically, the amount of tissue loss linearly correlated with total SCP parafoveal PD and the area of tissue loss colocalized with the region of SCP angiograms, showing the lowest parafoveal PD in all. In this context, it is important to notice how Muller cells mediate retinal neurovascular coupling and thus profoundly influence local retinal blood flow through the secretion of mediators such as nitric oxide and arachidonic acid metabolites, so much so that functional hyperemia does not occur when neuron-to-glia communication is interrupted [13]. In addition, Muller cells are a highly relevant source of VEGF and its receptors in the retina [21]. During the process of formation of LMHs, foveal cone Muller cells become damaged by anterior–posterior traction [22]. We theorize that a specific pattern of damage may give rise to critical Muller cell loss or disfunction, leading to a failure in maintenance of macular physiological capillary trophism and reactivity. At the same time, Muller cell damage results in a deficiency in their prosurvival effect on retinal neurons, mediated by metabolic support and secretion of neurotrophins such as brain-derived neurotrophic factor (BDNF), ciliary neurotrophic factor (CNTF) and Glial cell line-derived neurotrophic factor (GDNF) [23]. This factor, coupled with the reduction in microvascular blood flow supply, may contribute to the progression of neuronal tissue loss over time. Stable LMHs also showed a significantly higher SCP foveal VD. Compared to the general population, foveal VD was borderline to normal in stable LMHs and lower than normal in progressive LMHs both for SCP and DCP [24]. This could be the result of a longstanding lack of Muller cell trophic effects on retinal microvasculature of the fovea. Relevantly, according to our results LMHs showing progression of tissue loss also manifested VA loss during the follow up differently from eyes with morphologically unmodified LMHs. In addition, the speed of tissue loss linearly correlated with BCVA loss during the follow up in progressive LMHs. Once again, it is important to highlight how balanced VEGF secretion from Muller cells is fundamental for the homeostasis of the retinal pigmented epithelium and serves as an antiapoptotic signal for neuronal cells as well [23]. In this context, endogenous VEGF would thus be attributed a protective role against the progression of the disease, differently from the aggravating action held in other retinal diseases [25]. Moreover, this positive contribution would be caused by a different paracrine effect of endogenous VEGF that is not directly related to its pro-angiogenic effect. The limits of the study include the small sample size precluding the application of multivariate analysis, the lack of baseline OCT angiograms and the lack of additional information derived from fluorescein angiography. Moreover, it should be considered that VD and PD could be influenced by foveal shape. We hope that our findings may encourage further investigation in the topic.

## Figures and Tables

**Table 1:** Anamnestic and clinical characteristics of study groups. Quantitative variables are described as median and IQR in brackets. BCVA = best corrected visual acuity; CV = cerebral vasculopathy; IOP = intraocular pressure; SAH = systemic arterial hypertension; SE = spherical equivalent.

	TL Group	ST Group	*p*
Age	73.8 (6.4)	75.6 (6.8)	0.45
Sex	M = 7/14 (50%)	M = 3/14 (21.4%)	0.24
CV or dementia or previous stroke	3/14 (21.4%)	2/14 (14.3%)	0.99
Diabetes mellitus	2/14 (14.3%)	1/14 (7.2%)	0.99
SAH	8/14 (57.2%)	6/14 (42.9%)	0.46
BCVA at baseline (Decimals)	0.9 (0.12)	0.9 (0.05)	0.23
BCVA at last follow up (Decimals)	0.78 (0.09)	0.9 (0.1)	<0.001
Follow up duration (years)	2.16 (0.43)	2.37 (0.52)	0.88
SE (D)	−0.62 (1.34)	−0.44 (1.15)	0.75
Pseudophakia	10/14 (71.4%)	9/14 (64.3%)	0.68
IOP at baseline	14.4 (2.6)	15.1 (1.8)	0.67
IOP at last follow up	15.2 (2.3)	14.9 (2.1)	0.72

**Table 2:** Comparison between the two study groups on the basis of OCT-B scan and OCT-A variables of interest. CFZ = capillary free zone; DCP = deep capillary plexus; EZ = ellipsoid zone; LHEP = lamellar hole-associated epiretinal proliferation; PD = perfusion density; PVD = posterior vitreous detachment; SCP = superficial capillary plexus; ST = stable; TL = tissue loss; VD = vessel density.

		TL Group	ST Group	*p*
PVD	Complete	5/14 (35.71%)	6/14 (42.86%)	0.99
	Incomplete	7/14 (50%)	5/14(35.71%)	0.70
EZ disruption		3/14 (21.43%)	2/14 (14.28%)	0.99
LHEP		4/14 (28.57%)	0/14 (0%)	0.098
SCP	CFZ circularity	0.51 ± 0.07	0.60 ± 0.08	0.003
	CFZ area (mm^2^)	0.45 ± 0.11	0.40 ± 0.12	0.036
	CFZ perimeter (mm)	3.32 (0.36)	2.89 (0.24)	0.041
	Foveal VD (%)	16.35 ± 1.13	20.42 ± 1.78	0.012
	Foveal PD (%)	13.85 (1.18)	18.98 (1.6)	0.007
	Parafoveal VD (%)	0.44 ± 2.36	0.47 ± 2.13	0.071
	Parafoveal PD (%)	0.36 (2.77)	0.43 (2.83)	0.009
DCP	CFZ circularity	0.55 (0.09)	0.62 (0.07)	0.003
	CFZ area (mm^2^)	0.47 (0.18)	0.43 (0.12)	0.055
	CFZ perimeter (mm)	3.27 (0.22)	2.95 (0.27)	0.067
	Foveal VD (%)	19.21 ± 1.34	22.13 ± 1.59	0.087
	Foveal PD (%)	15.82 ± 1.69	20.10 ± 1.44	0.021
	Parafoveal VD (%)	0.47 ± 2.87	0.49 ± 2.52	0.147
	Parafoveal PD (%)	0.36 ± 2.11	0.45 ± 2.32	0.005

## Data Availability

Data are available on request.
